# Distinct long-term effects on lung function and airway remodeling in ovalbumin and house dust mite mouse models of experimental asthma

**DOI:** 10.1038/s41598-026-47822-x

**Published:** 2026-04-18

**Authors:** M. Andrea Markus, Jonas Albers, Frauke Alves, Christian Dullin

**Affiliations:** 1 Translational Molecular Imaging, Max-Planck-Institute for Multidisciplinary Sciences - City Campus, Hermann-Rein Str. 3, 37075 Goettingen, Germany; 2 Institute of Clinical and Interventional Radiology, University Medical Center Goettingen, Robert-Koch-Str. 40, 37075 Goettingen, Germany; 3 Present Address: European Molecular Biology Laboratory, Hamburg Unit c/o DESY, Notkestrasse 85, 22607 Hamburg, Germany; 4 Clinic of Hematology and Medical Oncology, University Medical Center Goettingen, Robert-Koch-Str. 40, 37075 Goettingen, Germany; 5 Department Diagnostic and Interventional Radiology, GermanCenterforLungResearch (DZL), University Hospital Heidelberg, Im Neuenheimer Feld 420, 69120 Heidelberg, Germany; 6 Translational Lung Research Center Heidelberg (TLRC), German Center for Lung Research (DZL), Im Neuenheimer Feld 130.3, 69120 Heidelberg, Germany; 7 Italian synchrotron “Elettra”, Strada Statale 14 - Km 163, 5 in AREA Science Park, Basovizza, Trieste, Italy

**Keywords:** Experimental asthma, OVA AAI model, HDM AAI model, X-ray-based lung function, Diseases, Immunology, Medical research, Physiology

## Abstract

Although mouse models of acute allergic inflammation (AAI) have long been used to study asthma mechanisms, their ability to reproduce human disease remains controversial and long-term studies of chronic asthma remain scarce. We compared lung function and tissue changes in three experimental asthma models after four months of recovery. Using x-ray–based lung function (XLF) analysis, we assessed lung parameters and diaphragm motion, alongside lung expression of α-smooth muscle actin and key extracellular matrix proteins, collagen and elastin. A severe ovalbumin (OVA) model (SAA) produced stronger acute and long-term effects than a milder OVA model (MAA), reflected in both lung dysfunction and tissue remodeling. Lung function remained impaired in SAA and in a house dust mite (HDM) model months after recovery. However, SAA and HDM showed distinct long-term remodeling: While SAA revealed reduced decay rate, collagen deposition and elastin reduction, HDM induced no histological abnormalities but resulted in smaller lung areas and greater diaphragm motion. This first direct comparison of long-term outcomes across AAI models highlights substantial heterogeneity that parallels differences seen in human asthma. These findings underscore the need for careful model selection in studies of chronic asthma and demonstrate the sensitivity of non-invasive XLF for detecting subtle changes.

**Supplementary Information:**

The online version contains supplementary material available at 10.1038/s41598-026-47822-x.

## Introduction

Allergic asthma is a heterogeneous inflammatory lung disease affecting millions of people worldwide, with a steadily increasing incidence^1^. Mouse models of allergic inflammation have been widely utilized to mimic this pathology, providing insights into underlying mechanisms and facilitating the development of therapeutic approaches. While much research focuses on acute asthma attacks, the findings of Gelb et al.^2^ suggest that patients with moderate to severe chronic persistent asthma experience a significant loss of lung elastic recoil, despite the absence of emphysema and ongoing optimal therapy. This challenges the prevailing notion that fixed airflow limitation in asthma is solely due to airway remodeling, indicating that additional factors, such as changes in lung tissue elasticity, may also play a critical role. To our knowledge, this aspect has not yet been explored in preclinical research, likely due to the known limitations of mouse models in replicating chronic allergic inflammation and the lack of sensitive imaging or measurement techniques.

One of the oldest and most widely used models is the ovalbumin (OVA)-induced acute allergic inflammation model, in which mice are sensitized with the protein antigen OVA combined with the pro-T-Helper cell (Th2) adjuvant aluminum hydroxide. Subsequent repetitive OVA instillations into the airways trigger a Th2-skewed adaptive immune response, leading to eosinophilic airway inflammation and airway hyperreactivity (AHR)^3^. A more clinically relevant model is the house dust mite (HDM)-induced model, which replicates key hallmarks of allergic asthma observed in humans, including not only AHR but also elevated circulating levels of total and HDM-specific IgE and IgG1^4^. While these models have provided valuable insights, they may not fully capture the complexity of human asthma^5^.

Modeling long-term effects of chronic asthma remains a significant challenge. More aggressive models tend to transition into a pulmonary fibrosis phenotype, whereas milder models often exhibit complete resolution of symptoms over time^6^. In this context, both acute and chronic allergen exposure models have been compared. Nials and Uddin^7^ emphasized the importance of selecting appropriate mouse models based on specific research objectives, acknowledging that while these models contribute valuable insights into asthma pathophysiology, they have inherent limitations in fully replicating the complexity of the human disease.

In this study, we compare the long-term effects in three different acute allergen exposure models of allergic airway inflammation: a severe OVA model (SAA)^3^, a mild OVA model (MAA)^8^, and an HDM model (HDM)^9^. We employ X-ray lung function (XLF) measurements based on cinematic 2D X-ray imaging (XLF)^10^ to assess functional changes during disease progression, comparing acute phase and recovery phase (121 days post-allergic reaction) between healthy, vehicle treated and allergen exposed animals. XLF leverages the modulation of X-ray transmission in the chest region by breathing and inflammation, allowing for the quantification of these aspects at very low radiation doses. This technique is particularly valuable for longitudinal in vivo studies and has been shown to be more sensitive than whole-body plethysmography, able to demonstrate treatment responses as well as airway hyperresponsiveness to methacholine challenge^10,11^. Specifically, the measurement of the temporal behavior of lung elastic recoil during the expiration phase enabled the measurement of a surrogate marker for lung elasticity in the severe OVA model of AAI^10^. Here, we applied this technique for the comparison of the long-term effects of three different AAI models and demonstrate distinct functional and morphological differences among these. The severe OVA model triggered stronger acute and lasting effects than the milder OVA model, driving greater lung dysfunction and remodeling. Long-term remodeling outcome between SAA and HDM diverged, with HDM showing no histological abnormalities but smaller lung areas and larger diaphragm motion. Our findings suggest that even acute allergen exposure mouse models, when adequately optimized, can reproduce key aspects of well-controlled chronic asthma observed in humans.

## Methods

### Allergic airway inflammation (AAI) mouse models

**Fig. 1:**
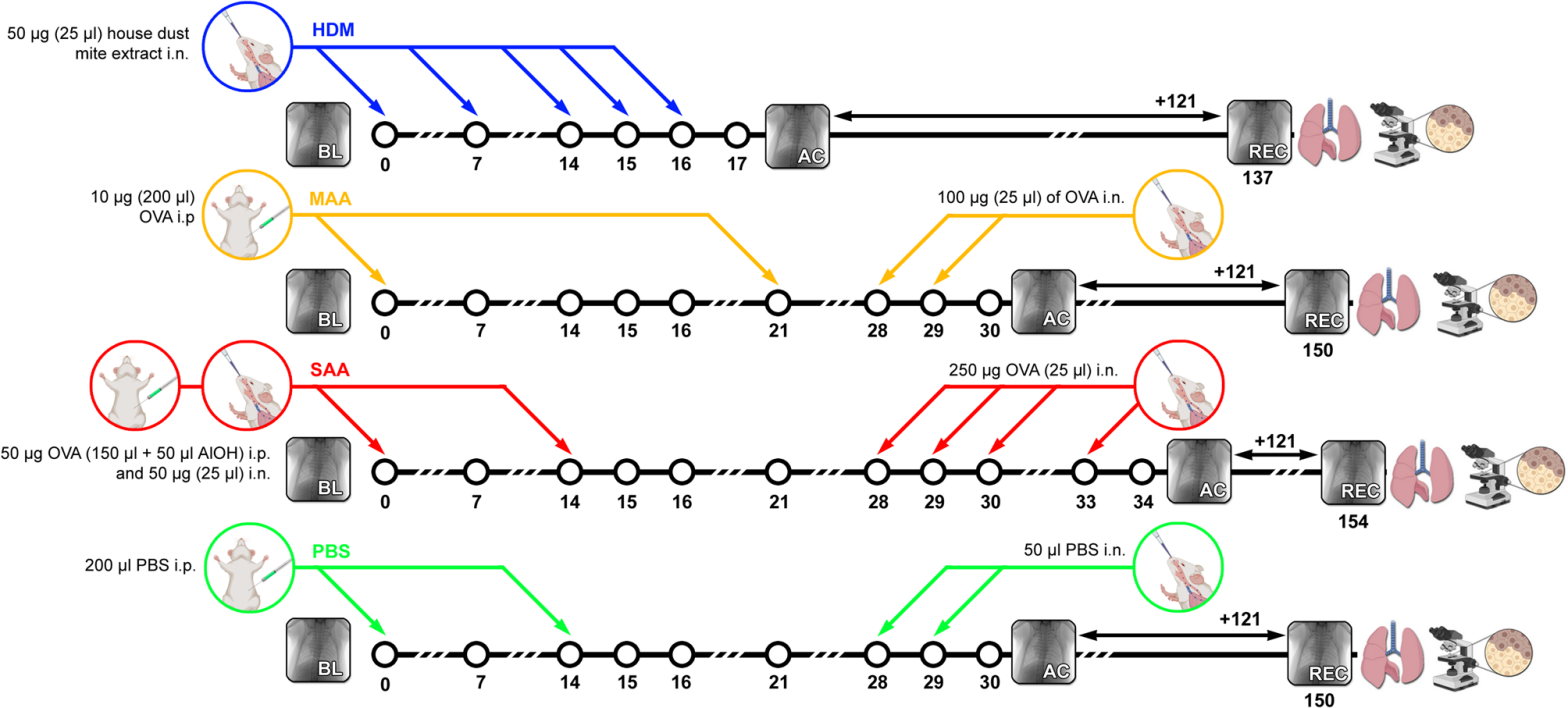
Scheme of the 3 AAI mouse models and PBS control, showing time points of allergen sensitization and challenges, as well as time points of XLF measurements (BL = baseline; AC = after challenge; REC = recovered). 121 days after the last challenge mice were euthanized and the lung excised for histological evaluation. i.n. = intranasal; i.p. = intraperitoneal.

Female BALB/c mice (8–9 weeks old) were purchased from Charles River Laboratories Inc. Mice were housed in a controlled environment with a regular 12-h dark: light cycle, at 22 °C and were fed laboratory chow (SAFE) and tap water ad libitum. Three models of experimental allergic airway inflammation (AAI) were generated, a mild (MAA, N = 7) and a severe (SAA, N = 7) ovalbumin (OVA) (Hyglos) induced AAI mouse model as well as a house dust mite (HDM) (N = 7) AAI mouse model. MAA mice received two immunizations by intraperitoneal (i.p.) injection of 10 µg (200 µl) OVA on days 0 and 21 and were challenged intranasally (i.n.) on days 28 and 29 with 100 µg (25 µl) of OVA. SAA mice were sensitized twice with 50 µg OVA (150 µl + 50 µl AlOH) i.p. and 50 µg (25 µl) i.n. on days 0 and 14 and were then challenged 4 times on days 28, 29, 30, and 33 with 250 µg OVA (25 µl) i.n.. HDM mice received 5 i.n. instillations of 50 µg (25 µl) house dust mite extract (Greer Laboratories) on days 0, 7, 14, 15 and 16. Both OVA and the HDM extract were diluted in phosphate-buffered saline (PBS). Control mice either received PBS (PBS, N = 7) or were left untreated (healthy, N = 7). All groups were then kept without any additional treatment for 121 days (Fig. 1). Group size was calculated by a power analysis based on our previous experiments^10,11^. No animals were excluded from the original cohort. Mice were randomly assigned to the different AAI models. The study was performed in a non-blinding manner. All animal in vivo procedures were performed in compliance with the guidelines of the European (2010/63/EU) and the German ethical laws and were approved by the administration of Lower Saxony, Germany (Nr. G15.1747). The study was reported in accordance with ARRIVE guidelines.

### XLF—non-invasive low dose x-ray based planar cinematic lung function measurement

**Fig. 2:**
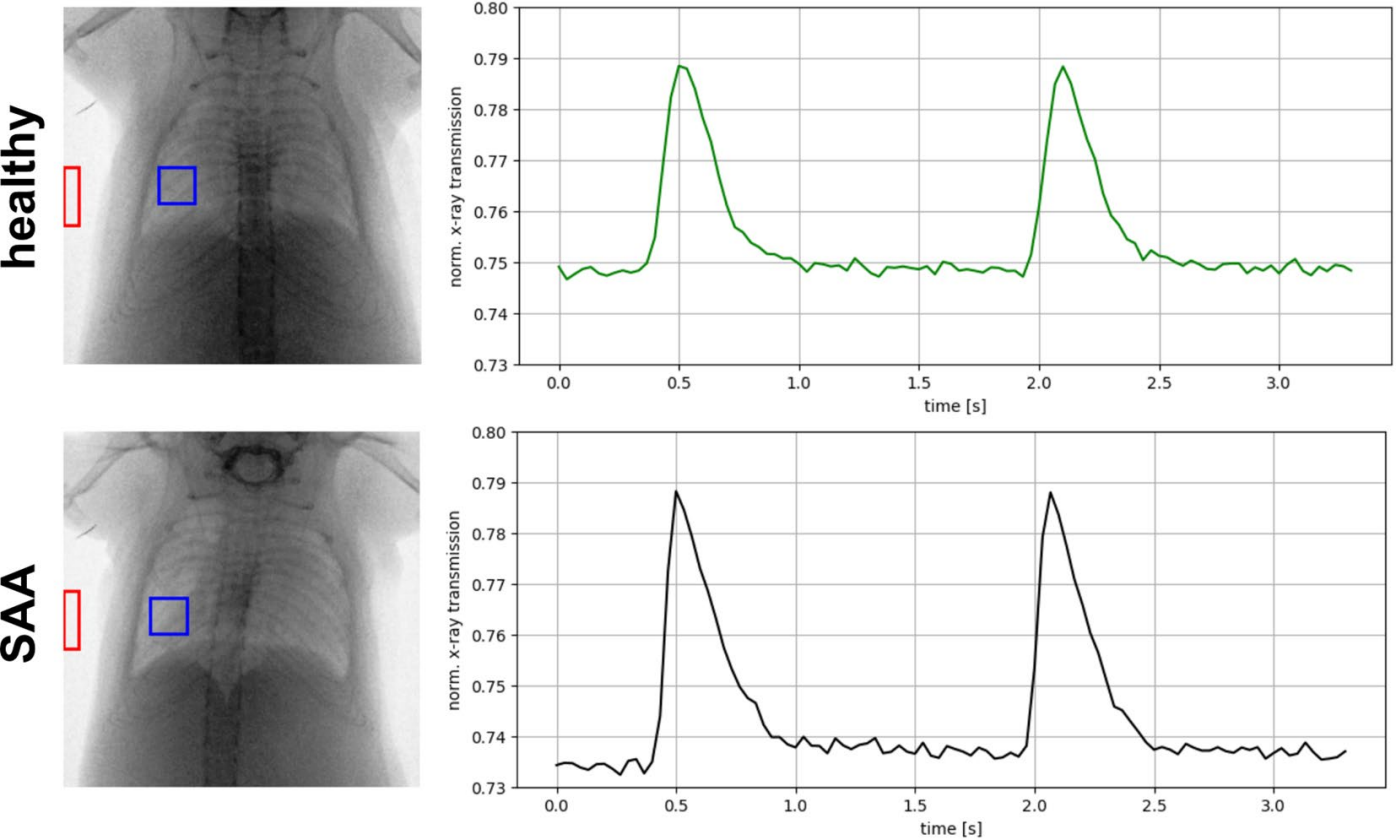
Principle of lung function measurement by low dose 2D cinematic x-ray imaging. X-ray movies of the chest region were recorded for 34 s at 30 fps and *≈*6 mGy. The average X-ray transmission over time was measured in a lung region (blue square) and normalized to a background region (red square), yielding the curves shown (only two breathing events are displayed). Notably, the healthy mouse exhibits a distinctly different curve compared to the SAA mouse in the acute phase of the disease (48 h post-challenge). These curves were parameterized by fitting a quadratic exponential decay function to the decay phase.

XLF was performed as previously described^10–12^ by acquiring planar cinematic x-ray images of the chest of unrestrained but isoflurane anesthetized mice using a low dose in vivo microCT (QuantumFX, Perkin Elmer). In brief, mice were positioned on the microCT bed, and their respiratory rate was controlled by adjusting the isoflurane concentration (1.5–3%) to maintain a breathing frequency of approximately 0.7 Hz. Radiographic images were acquired using an X-ray tube voltage of 90 kV and a tube current of 100 µA. The CT system produces a movie from a series of 2D radiographs that track chest movement and x-ray transmission through the thoracic region during active breathing. Regions of interest (ROIs) are placed on the left and right lung lobes at the time of maximal chest contraction, as well as in a background area. X-ray transmission over time is calculated as the average intensity within the lung ROIs, normalized to the average intensity of the background ROI to compensate for short-term fluctuations in x-ray output. The resulting function is dimensionless. To correct for long-term drift in x-ray production, a slowly varying background component is removed. Movies were recorded for 34 s, capturing approximately 21 breathing cycles per mouse. Each breathing event is parameterized, and the mean across all cycles within the recording period is used to characterize lung function for each animal. The principle of XLF is shown in Fig. 2. During expiration a quadratic exponential decay curve (Eq. 1) was fit to measure how quickly the lung deflates, reported as the decay rate (k).

1 $$f(t) = a \cdot e^{{ - kt^{2} }} + b$$

### Diaphragm motion analysis

Diaphragm motion analysis was performed as we described before^10^, by calculating the average and the standard deviation (*Std*) of the brightness per pixel over time in the acquired x-ray movies. As the motion of the diaphragm causes the movement of the lower border of the lung it can be detected as an area of a strong Std. The width of this area represents the average diaphragm movement (*s*_*dia*_). Because the duration of the breathing event is shorter than the periods between the breathing events, the average of the x-ray transmission over time predominantly depicts the size of the lung in exhalation (*A*_*Lung*_).

### Bronchoalveolar lavage (BAL)

Following XLF on day 121 after the final challenge, mice were euthanized with an overdose of isoflurane, followed by immediate tracheotomy. Bronchoalveolar lavage (BAL) was performed by gently washing the airways three times with 500 µl of 2% FCS/PBS after exposing and cannulating the trachea. The collected lavage volumes were pooled and washed once in the same buffer. Recovered cells were counted using a hemocytometer, and 3 × 10^4^ cells were used to prepare cytospins, which were subsequently stained with Giemsa (Sigma Aldrich) for differential cell counting under a light microscope.

### Histology of lung sections

Following the bronchoalveolar lavage, the lungs were excised, fixed in 10% buffered formalin and embedded in paraffin. Three µm-thick paraffin lung sections containing main stem bronchi were used for staining procedures and subsequent imaging by light microscopy with an Axioskop 2 microscope (Leica Microsystems). Periodic-acid Schiff (PAS) staining was performed according to the manufacturer’s protocol (Merck). Collagen was stained with a Masson trichrome kit (Sigma Aldrich) to assess airway fibrosis. Sections were microscopically scored from 0 to 3 in a blinded fashion by three observers to evaluate the amount of mucus producing goblet cells (designated PAS-score) and the amount of collagen in the Masson trichrome staining (MT score), with 0 = no mucus and no collagen, 1 = low mucus and collagen, 2 = medium mucus and collagen, 3 = high mucus and collagen, as described before^10^. An Elastica van-Gieson kit (Morphisto) was used for staining of elastic fibers. The amount of elastin was measured on 3 snap shots per section using a threshold and calculated as a ratio to the lung tissue area. Alpha-smooth-muscle actin (*α*SMA) was stained by anti-mouse-*α*SMA antibody (Abcam, ab5694, 1:500). *α*SMA expression was quantified in the following way: 2–6 small (< 8 µm^2^), medium (9–20 µm^2^) and large bronchi (> 20 µm^2^) were selected on stitched images, the epithelial cells surrounding a bronchus was segmented based on its color (threshold) and the ratio to the area of the bronchus was calculated.

### Data processing and statistics

Because group sizes were unequal and variance homogeneity could not be assumed, pairwise group comparisons were performed using the Brunner–Munzel test, a non-parametric procedure that does not require identical distribution shapes or equal variances between groups and provides a robust test of stochastic dominance. To account for the large number of pairwise comparisons while maintaining adequate statistical power, *p*-values were corrected for multiple testing using the Benjamini–Hochberg false discovery rate (FDR) procedure. This approach controls the expected proportion of false positives among significant results and is well suited for exploratory biological data involving multiple related group comparisons. The XLF algorithm is implemented in Matlab 2011b (7.13.0.564).

## Results

To compare the long-term effects of different mouse models of allergic inflammation, we assessed the lung function of untreated (healthy), vehicle treated (PBS) and the different allergen treated (SAA, MAA, HDM) mice directly after challenge (acute allergic phase, ac), then let the mice recover for about 4 months before measuring lung function again (recovered phase, rec) (Fig. 1).

### XLF analysis reveals long-term decay in lung function of SAA and HDM mouse models

**Fig. 3:**
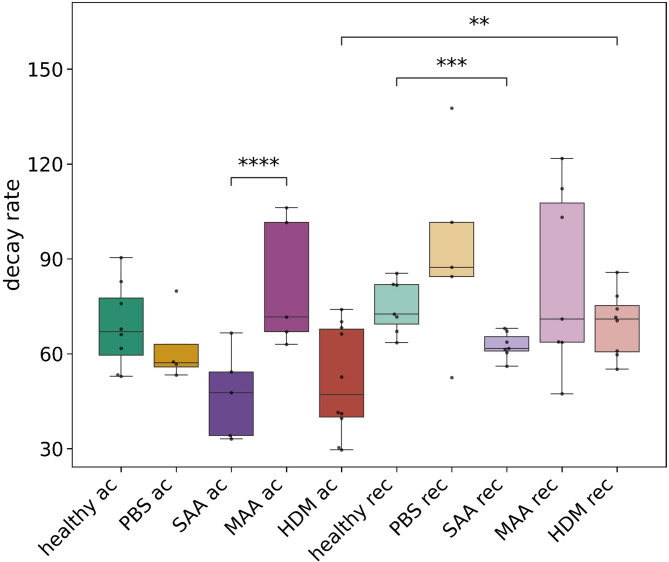
Quantitative comparison of the calculated decay rate in the fitted quadratic decay functions, measured by XLF. The acute asthmatic mice, particularly those in the SAA-ac group, exhibited a reduction in the time constant, indicating a prolonged expiration phase, and were significantly different to the MAA-ac mice. No significant differences were observed between the decay rates of HDM-ac and SAA-ac groups. The PBS-ac group also demonstrated a slightly reduced decay rate. During the recovery phase, SAA-rec mice showed a significant reduction in the decay rate, though the decrease was less pronounced compared to the acute phase. *p*-value definitions: ** < 0.01; *** < 0.001; **** < 0.0001.

X-ray-based lung function (XLF), a simple non-invasive low-dose 2D cinematic x-ray imaging procedure was performed to assess lung function (Fig. 2). In XLF imaging, the average X-ray attenuation over the chest area is continuously monitored, reflecting the respiratory cycle of the mouse. During the expiratory phase, particularly the descending limb of each breathing event, a quadratic exponential decay function is fitted to quantify the rate of lung deflation, defined as the decay rate. When compared to healthy mice, XLF showed a reduced decay rates for both SAA and HDM at the acute phase and to similar degrees, albeit non-significant (Fig. 3). Interestingly, also the PBS treated group showed a small reduction in the decay rate (Fig. 3). MAA—the mild OVA induced allergic airway inflammation model—showed no significantly different decay rate compared to the control group at the acute AAI stage.

When comparing the decay rates (Fig. 3) between the recovered phase (121 days after the last challenge) with the acute phase (*≈* 48 h after the last challenge), SAA shows a smaller but still significant reduction in the decay when compared with the healthy controls. The HDM model has “recovered” and is not different to healthy controls. At the recovered phase, the PBS treated group had regained the same decay rate like the control group.

### Diaphragm motion analysis reveals significant long-term effects on lung volume of HDM mice

**Fig. 4:**
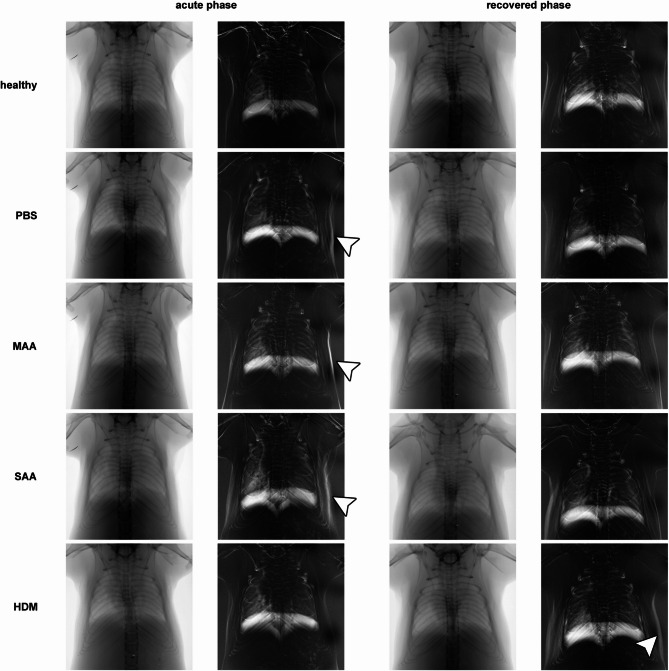
Representative diaphragm motion analysis in acute and recovered phase in different AAI mouse models. From the low-dose 2D cinematic X-ray acquisitions, both the average and the standard deviation over time were calculated. Due to the longer expiration phases compared to inspiration phases under isoflurane anesthesia, the averaged images predominantly capture the lung shape during expiration. The standard deviation images highlight areas of the mouse that moved. While most of the breathing motion was driven by diaphragm movement, particularly in the acute phase of the asthma models, activation of the intercostal muscles was also observed (arrow heads), suggesting a forced expiration pattern. Interestingly, this same effect was seen in the PBS-treated mice, indicating ongoing acute lung inflammation. In the recovery phase, this behavior was less pronounced. Lung area during expiration (*A*_*Lung*_) and the average width of the diaphragm’s traveling range (*s*_*dia*_) were quantified.

**Fig. 5:**
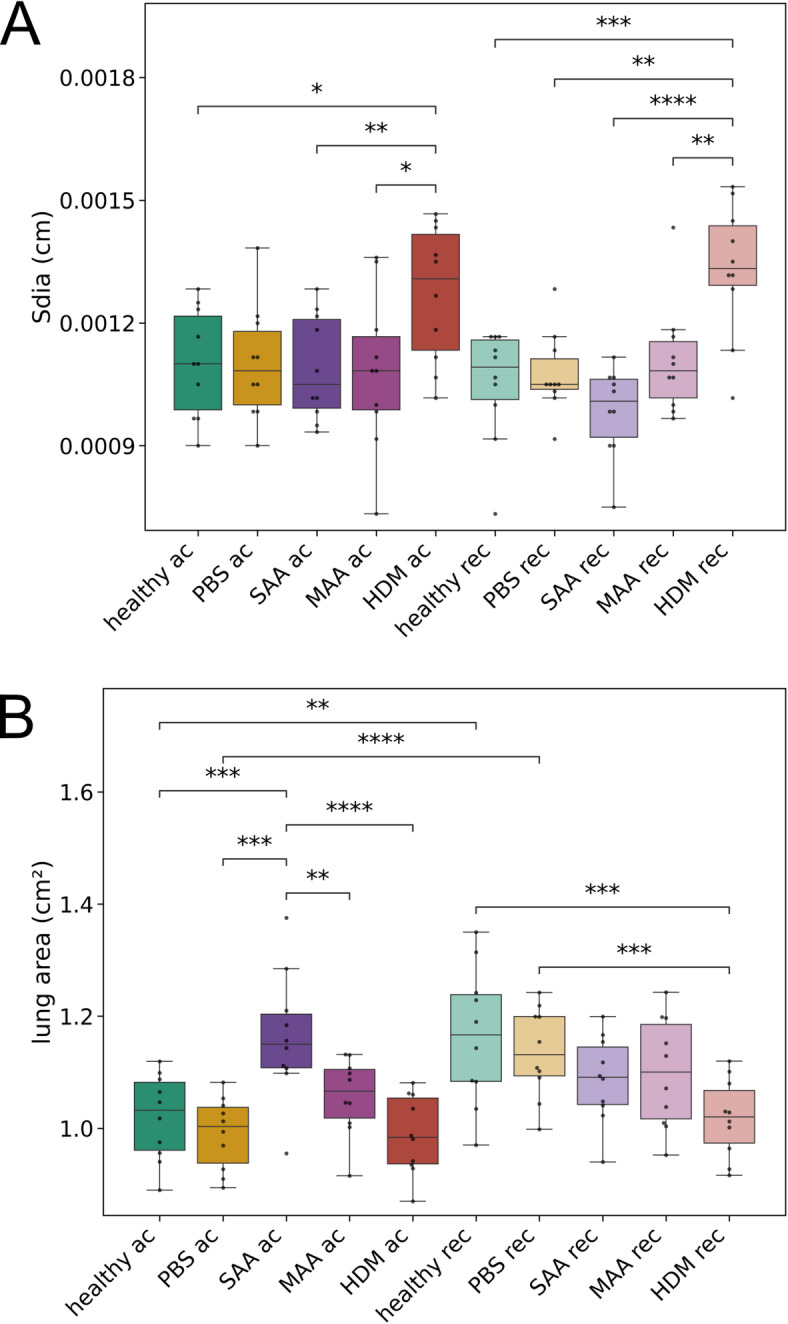
DMA reveals significant but different long-term changes in the lung area and diaphragm motion for the three AAI models in acute (ac) and recovered (rec) phase. (**A**) Extent of diaphragm motion calculated as width of the diaphragm traveling range in the standard deviation of the XLF movies (*s*_*dia*_). (**B**) Lung area calculated from XLF movies, indicative of the lung volume at exhalation. *p*-value definitions: * < 0.05; ** < 0.01; *** < 0.001; **** < 0.0001.

Diaphragm motion analysis (DMA) was performed by calculating the average and standard deviation over time from the acquired XLF movies (Fig. 4), allowing for the measurement of the lung area during expiration as well as the traveling range of the diaphragm. In agreement with our earlier results^10^, diaphragm motion distance (*s*_*dia*_), as measured from the standard deviation images, was not significantly different between acute SAA-ac and control groups (healthy-ac and PBS-ac) (Fig. 5A). However, the acute HDM-ac model exhibited greater diaphragm motion distances than all other groups (Fig. 5A). Moreover, even after 121 days of recovery, HDM-rec mice demonstrated significantly increased diaphragm movement than healthy-rec mice or any other AAI models. While most of the breathing motion was driven by diaphragm movement, activation of the intercostal muscles was also observed, visible by higher standard deviation of the x-ray attenuation along the side of the ribs, (Fig. 4, arrow heads), suggesting a partially forced expiration pattern. This was the case particularly in the acute phase of all asthma models, but also persisting long-term in the HDM-rec model.

In terms of changes in lung area, DMA revealed significant long-term differences among the three AAI models. The lung area measured by this method reflects the area during exhalation and is indicative of lung volume at this breathing state. In the acute phase of AAI, SAA-ac showed a significantly larger lung area than the controls (healthy-ac and PBS-ac, Fig. 4 and Fig. 5), consistent with our earlier DMA results for this AAI model^10^. MAA-ac and HDM-ac did not show significant differences in lung area when compared to the controls. After 121 days of recovery, lung area in the SAA-rec mice returned to levels similar to the controls. Surprisingly, however, lung area during exhalation at this time point was significantly lower in HDM-rec mice compared to healthy-rec mice.

Since the lung area during expiration serves as a surrogate marker for diaphragm position—reflecting an equilibrium between the respiratory muscles and the elastic recoil of the lung—it can be speculated that in HDM-rec mice, the lung tissue has become stiffer, while in the SAA-rec mice, the tissue remains less elastic.

### Histology confirms differential long-term tissue remodeling in dependence of the AAI model

Following sacrifice of the mice after a 121-day recovery period from the acute antigen challenge, we performed a panel of histological stainings of lung paraffin sections in an attempt to explain the differences in lung function and diaphragm motion that we observed between the OVA and HDM models and correlate the data to morphological alterations. PAS staining showed that none of the lungs of any group contained mucus or hyperplasia of goblet cells, confirming the resolution of the acute inflammation 121 days after the last challenge (data not shown). Furthermore, BAL analysis showed that none of the lung lavages from any of the examined groups contained eosinophils, which typically increase during the acute phase of the allergic reaction (Suppl.Fig. S1).

**Fig. 6:**
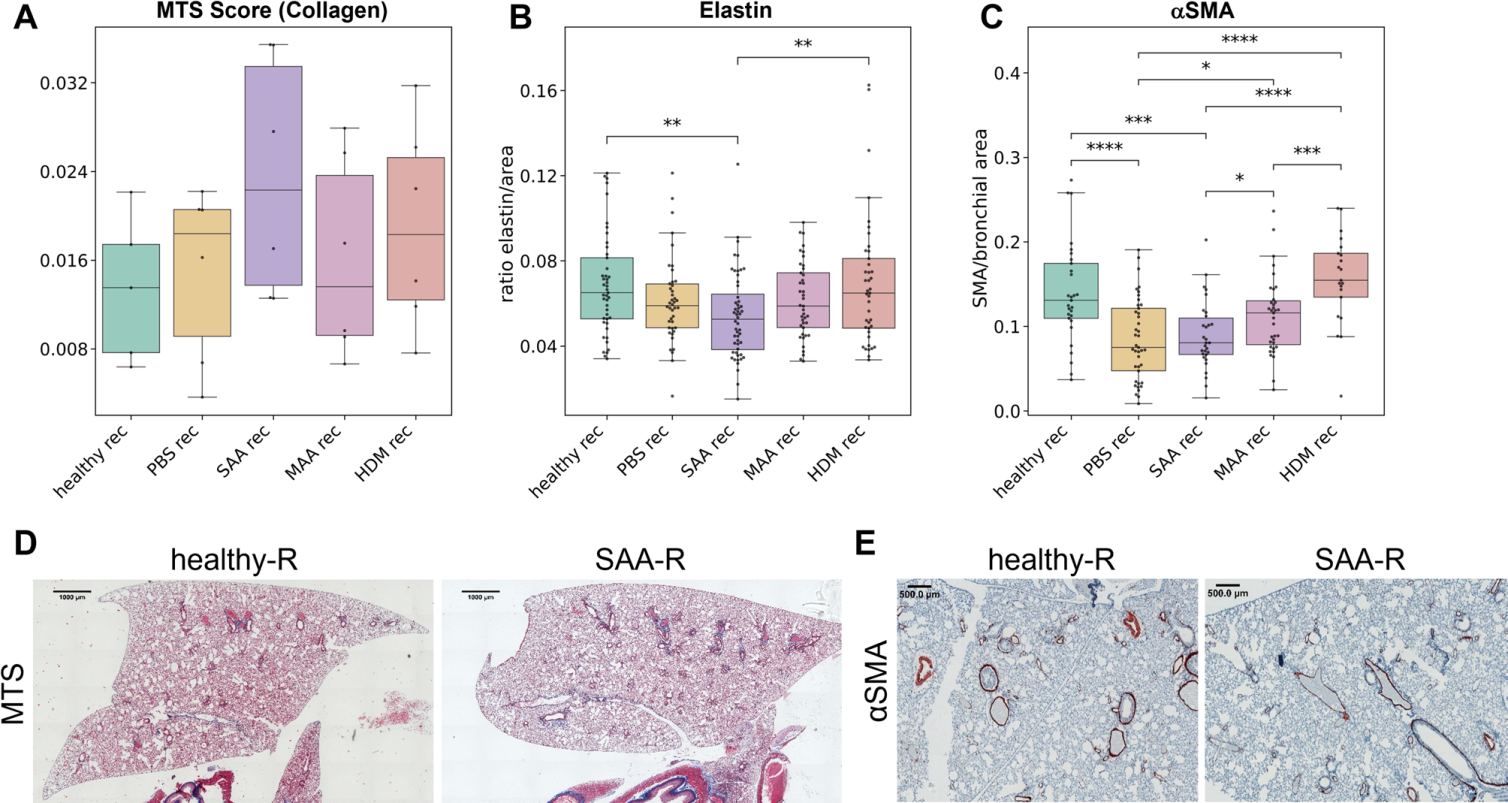
Histology reveals different effect on long-term tissue remodeling in dependence of the AAI model used. Stainings were performed on lung paraffin sections of the 3 AAI models SAA, MAA and HDM, as well as the controls healthy and PBS, at 121 days after the last antigen challenge (rec for recovered). (**A**) Amount of collagen determined by MT score. (**B**) Elastin expression determined as the ratio of elastin fibers per lung area. (**C**) *α*SMA expression determined as ratio of *α*SMA positive area to bronchial area. (**D**) Representative images of MTS-stained tissue sections from a healthy-rec and a SAA-rec lung lobe, showing a higher collagen amount (blue staining) in SAA-rec than in healthy-rec. (**E**) Representative images of sections from a healthy-rec and a SAA-rec lung lobe stained with an anti-*α*SMA antibody (brown staining, arrows), showing decreased expression of *α*SMA around bronchioles in the SAA-rec lung section compared to healthy-rec controls. *p*-value definitions: * < 0.05; ** < 0.01; *** < 0.001; **** < 0,0001.

Masson trichrome staining (MTS) was performed to evaluate the amount of fibrosis-related collagen at the recovered time point. The results displayed a tendency towards a higher amount of collagen in the lungs of the SAA-rec and HDM-rec groups when compared to controls (healthy-rec and PBS-rec) and to the weaker OVA-induced AAI model MAA-rec, but did not reach significance (Fig. 6A, D).

Staining of elastin fibers, revealed significantly reduced amounts in SAA-rec lungs compared to healthy-rec controls (Fig. 6B), confirming our earlier findings^10^ and suggesting that the severe OVA-induced AAI leads to a persistent loss of elasticity of the lung, even at 121 days of recovery from the acute inflammation. The weak OVA model MAA-rec and the HDM-rec model did not show any long-term difference in elastic fibers when compared to controls (Fig. 6B).

Anti-*α*SMA staining also confirmed our earlier findings with the severe OVA model SAA-rec^10^, showing that this model leads to a long-term loss of *α*SMA around bronchi and bronchioles (Fig. 6C, E). A reduction in *α*SMA was also found with the mild OVA model MAA-rec, albeit not as pronounced as in SAA-rec mice (Fig. 6C). HDM-rec did not show any difference in *α*SMA when compared to healthy-rec animals (Fig. 6C). A surprising but important outcome was the finding that the PBS challenges also led to a significant loss of *α*SMA after 121 days of recovery (Fig. 6C). Moreover, both MTS and elastin staining of PBS-rec lungs showed values similar to those found in the weak OVA model MAA-rec, with elevated MT Score and decreased elastin, although both were not significantly different from healthy-rec controls (Fig. 6A, B). These data however suggest, that PBS or liquid instillation in the lung leads to remodeling of the lung and has therefore limited use as a vehicle control.

## Discussion

To evaluate long-term effects in experimental asthma, we conducted a combined functional and histological analysis of three different acute allergen exposure models of allergic airway inflammation in mice: severe ovalbumin-induced (SAA)^3^, mild ovalbumin-induced (MAA)^8^, and house dust mite-induced (HDM)^9^. We compared functional and morphological parameters of the lung in the acute phase of the disease with those in the recovered phase, defined as 121 days after the last allergen exposure. Our findings revealed a comparable, albeit non-significant, reduction in lung recoil in the acute phase for both, the SAA and HDM model, consistent with a loss of lung function found also by other more invasive techniques^13,14^, whereas MAA mice showed no reduction.

Surprisingly, the PBS control group also exhibits a slight decline in elastic recoil, suggesting that PBS or the instillation of liquid in the lung per se may lead to an inflammatory response. While no evidence is found in the literature on PBS induced cell changes and no significant change was found in the number of immune cells in our own BAL examinations (data not shown), we speculate that PBS may cause a mild inflammatory reaction that can result in a degree of lung remodeling. As we found a similar effect in previous XLF experiments (data not shown), we included healthy mice as additional control group. Interestingly, even after 2 months recovery, *α*SMA expression was found significantly reduced in the PBS group compared to the healthy controls. Therefore, PBS itself and/or the instillation of liquid in the lung may interfere with the disease progression, which we believe should be considered in such experiments.

In the recovered phase, reduction in elastic recoil persisted in SAA and HDM, indicating long-term structural alterations, consistent with long-term effects on lung function seen in asthmatic humans^15^. Diaphragm motion analysis revealed notable differences between HDM and SAA in both the acute and recovered phases. In the recovered phase, HDM mice exhibit a decreased projected lung area and stronger diaphragm motion compared to SAA, suggesting fibrotic remodeling. These functional findings were supported by histological analysis, which showed an increase in collagen content in both HDM and SAA in the recovered phase, albeit this did not reach significance for HDM. However, at this late time point only the SAA model reveals a significantly reduced elastin content, suggesting that the loss of elastin may have overcompensated for the stiffening effect of collagen enrichment. The observed reduction in *α*SMA levels around bronchioles in PBS, SAA, and MAA mice may indicate a reversal of fibrotic progression in the recovered phase, whereas in lungs of HDM mice, *α*SMA expression levels remained at a similar level as healthy controls.

Understanding the remodeling of collagen and elastin in respiratory diseases is crucial for developing targeted therapies. For instance, therapies that inhibit excessive collagen deposition or promote elastin repair could potentially restore normal lung function. Moreover, biomarkers related to collagen and elastin turnover might serve as diagnostic or prognostic tools in managing respiratory diseases^16^. An early study concludes that elastinolysis occurs in the airways of asthmatic patients, possibly as a result of repair mechanisms triggered by chronic inflammation. Additionally, mechanical stretching induced by breathing and edema may contribute to the fragmentation of fibers in asthmatic airways. These structural changes in elastic fibers could play a role in the pathophysiology of asthma, potentially affecting airway elasticity and function^17^. A key study by Andersson et al. examined bronchial and alveolar tissue samples from asthmatic patients with varying levels of disease control. They found increased alveolar collagen I deposition in well-controlled asthmatic patients. In contrast, collagen VI was elevated in uncontrolled asthma^18^. These findings suggest that even with symptom control, structural remodeling continues in specific extracellular matrix (ECM) compartments. The amount and ratio of collagen and elastin is controversial. A more recent study reported that airway elastin is increased in severe asthma and relates to proximal wall area^19^. However, Godfrey et al. studied mild, mostly well-controlled asthmatics and found no significant difference in either collagen or elastin content in subepithelial regions of bronchial biopsies compared to controls^20^. Moreover, Antunes et al. found in a mouse model of chronic asthma, that both collagen and elastin increased in parallel after long-term allergen exposure^21^. These findings suggest that the ratio of collagen and elastin in mild and well-controlled asthma is preserved, even though total ECM content is increased, while in severe asthma, only elastin accumulates, disrupting the collagen elastin ratio.

Based on our BAL data, that showed a total absence of eosinophils in all groups (Suppl.Fig. S1), the 121 days post last challenge time point can indeed be considered a recovered state. Additionally, the fact that the total cell counts are quite comparable suggests that the acute immune reaction has entirely resolved. Therefore, the differences observed in motion analysis and histology must be attributed to variations in disease progression during the acute phase.

Finally, our study only uses female mice, which leaves open the possibility of sex-specific differences in lung function response to different allergens. We have previously examined the effect of sex on OVA induced lung function changes by XLF and have indeed found sex-dependent differences which we however attributed to the generally increased weight/size of male mice of the same age (Suppl. information in^11^). Recently significant sex-specific differences have also been reported in airway epithelial barrier function and responses to chronic injury^22^. XLF may however not be sensitive enough to disentangle sex-based differences from differences in allergen response and would therefore also not be able to distinguish sex-specific allergen induced lung remodeling.

To our knowledge this is the first comprehensive comparison of the long-term effects of different AAI mouse models and their assessment by x-ray-based lung function measurement. It is noteworthy that the different models exhibit such distinct long-term functional and morphological alterations. The SAA model (at least as it is used in our study) displays features similar to those of well-controlled chronic asthma patients^23^—such as the absence of eosinophils and a less fibrotic phenotype than HDM induced AAI—yet still shows reduced lung function. Thus, the SAA model may be useful for simulating treatment responses in such patients and could serve as a valuable tool for advancing drug development aimed at improving lung function in chronic asthma patients. Furthermore, our non-invasive low-dose X-ray in-vivo functional assessment has proven sensitive enough to detect these mild differences in lung function, which previously could only be identified through histology or invasive terminal in vivo techniques.

## Conclusion

In conclusion, our work shows that commonly used AAI mouse models, such as OVA and HDM induced models, lead to very different long-term effects on lung function and tissue remodeling which can be captured with our sensitive low-dose x-ray-based lung function technique. Moreover, we suggest that PBS and/or liquid instillation into the lung provoke changes in lung function parameters as well as the expression of *α*SMA and thus limits its use as control.

## Supplementary Information

Below is the link to the electronic supplementary material.

Supplementary Material 1

## Data Availability

Data can be obtained from the corresponding authors on reasonable request.
