# Supplementary material for: Distinct long-term effects on lung function and airway remodeling in ovalbumin and house dust mite mouse models of experimental asthma
Source: Sci Rep. 2026 Apr 18;16:12737. doi: 10.1038/s41598-026-47822-x (PMC13091909; doi:10.1038/s41598-026-47822-x)
Supplement: Supplementary file 1 — Supplementary Material 1 [file 41598_2026_47822_MOESM1_ESM.docx]

**Supplemental Material**

**
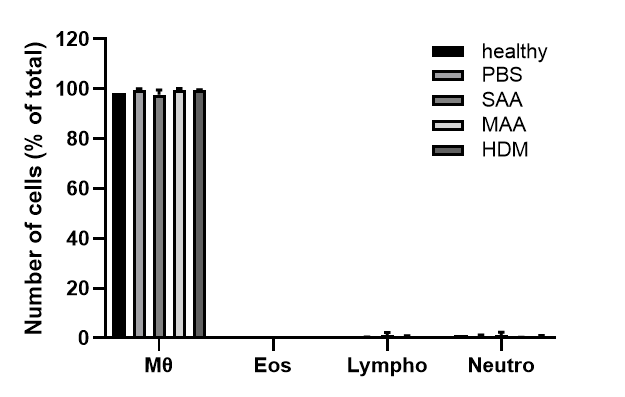
**

**Figure S1.** Bronchoalveolar lavage (BAL) shows no eosinophils in recovered animals, indicating resolution of the acute inflammation. Number of different immune cells (Macrophages-M*θ*, eosinophils-Eos, lymphocytes-Lympho and neutrophils-Neutro) shown as percentage of total cells extracted from BALs of the different AAI models (SAA, MAA, HDM) and controls (healthy, PBS) at the recovered timepoint.
